# Anti-Gastritis and Anti-Lung Injury Effects of Pine Tree Ethanol Extract Targeting Both NF-κB and AP-1 Pathways

**DOI:** 10.3390/molecules26206275

**Published:** 2021-10-16

**Authors:** Seung A Kim, Jieun Oh, Se Rin Choi, Choong Hwan Lee, Byoung-Hee Lee, Mi-Nam Lee, Mohammad Amjad Hossain, Jong-Hoon Kim, Sarah Lee, Jae Youl Cho

**Affiliations:** 1 Department of Integrative Biotechnology, Sungkyunkwan University, Suwon 16419, Korea; Seung-a26@naver.com (S.A.K.); martia96@gmail.com (J.O.); 2 Department of Bioscience and Biotechnology, Konkuk University, Seoul 05029, Korea; csr0701@gmail.com (S.R.C.); chlee123@konkuk.ac.kr (C.H.L.); 3 National Institute of Biological Resources, Environmental Research Complex, Incheon 22689, Korea; dpt510@korea.kr; 4 Department of Hospitality and Culinary, Ansan University, Ansan 15318, Korea; hhong3159@naver.com; 5 Department of Veterinary Physiology, College of Medicine, Chonbuk National University, Iksan 54596, Korea; mamjadh2@gmail.com

**Keywords:** *Pinus densiflora* Siebold and Zucc, ethanol extract (Pd-EE), anti-inflammation, gastritis, acute lung injury

## Abstract

An ethanol extract (Pd-EE) of *Pinus densiflora* Siebold and Zucc was derived from the branches of pine trees. According to the Donguibogam, pine resin has the effects of lowering the fever, reducing pain, and killing worms. The purpose of this study is to investigate whether Pd-EE has anti-inflammatory effects. During in vitro trials, NO production, as well as changes in the mRNA levels of inflammation-related genes and the phosphorylation levels of related proteins, were confirmed in RAW264.7 cells activated with lipopolysaccharide depending on the presence or absence of Pd-EE treatment. The activities of transcription factors were checked in HEK293T cells transfected with adapter molecules in the inflammatory pathway. The anti-inflammatory efficacy of Pd-EE was also estimated in vivo with acute gastritis and acute lung injury models. LC-MS analysis was conducted to identify the components of Pd-EE. This extract reduced the production of NO and the mRNA expression levels of iNOS, COX-2, and IL-6 in RAW264.7 cells. In addition, protein expression levels of p50 and p65 and phosphorylation levels of FRA1 were decreased. In the luciferase assay, the activities of NF-κB and AP-1 were lowered. In acute gastritis and acute lung injury models, Pd-EE suppressed inflammation, resulting in alleviated damage.

## 1. Introduction

In the human body, there is a defense mechanism that can protect the body from external attacks. External stimuli include organisms such as bacteria and fungi as well as toxins, fine dust, and disturbances that occur during biological activities. The representative defense system in our body is the inflammatory mechanism [1]. An inflammatory reaction refers to the process of fighting external stimuli by representative phagocytes waiting inside when external attacking factors penetrate barriers such as the skin and mucous membranes. This immune response is of great importance for maintaining homeostasis [2]. When an acute inflammatory response occurs from a few minutes to several hours to several days after a specific injury, inflammatory cytokines, chemokines, and proteins are expressed, and macrophages and neutrophils migrate to resist external invasion [3]. However, these reactions must start and end in a balanced way. Otherwise, tissue and body organs may be damaged, which may even lead to disease or cancer [4]. Therefore, research to discover herb extracts that can regulate inflammation responses and that are non-toxic is meaningful.

The innate immune response of the human body begins with the recognition of pathogens. Pattern recognition receptors (PRRs) play an important role in the cognitive process. The PRRs, which are proteins in the immune cells, can recognize molecules possessed by pathogens [5]. The conserved structures are pathogen-associated molecular patterns (PAMPs). Once PAMPs engage, PRRs send signals to intracellular cascades that increase the expression of many proinflammatory molecules [6]. Various ligands and receptors exist to induce inflammation. Among them, one representative compound is lipopolysaccharide (LPS), a characteristic component of the cell walls of Gram-negative bacteria and toll-like-receptor 4 (TLR4). After LPS engagement with TLR4, it conducts signaling to the two major downstream adaptor proteins, myeloid differentiation primary response 88 (MyD88) and toll/interleukin-1 receptor-domain-containing adapter-inducing interferon-β (TRIF) [7]. The adaptors initiate the translocation of nuclear factor kappa B (NF-κB) and activator protein-1 (AP-1) into the nucleus, changing their binding ability through activation-related pathways [8]. NF-κB and AP-1 control the transcription of genes to regulate the inflammation and homeostasis of the immune system [9,10]. When these pathways are activated, the cascades induce the release of pro-inflammatory cytokines such as inducible nitric oxide synthase (iNOS), cyclooxygenase-2 (COX-2), and tumor necrosis factor-α (TNF-α) [11]. The important point here is that if the inflammatory reaction, which is a defense mechanism, occurs for a long time or excessively, it will rather harm the body as an excessive immune reaction. This can lead to altered homeostasis, damage to tissues and organs, the development of diseases, and the progression of cancers [12]. Therefore, focusing on controlling inflammatory reactions is critical to health and quality of life. Currently, there are various types of drugs that can control excessive inflammation by suppressing inflammatory crises [13]. These immunosuppressants should have the maximum inhibitory effect at the minimum amount, but with minimum side effects. Studying natural plant extracts as ingredients in immunosuppressants can satisfy these conditions. Thus, herbal extracts as medicinal materials have attracted interest from researchers [13].

*Pinus densiflora* Siebold and Zucc. (Pinaceae family) is a pine tree that is evergreen, growing high, and long lived. Due to the features of the plant, this plant has been traditionally used for long time. In North America and in Europe, Scots pine trees are intaking as herbal tea with benefits to treat rheumatism, wounds, common colds, and pulmonary tract mucus lining inflammation [14]. According to the Donguibogam (Principles and Practice of Eastern Medicine) written by Jun Heo [15], it is known that pine resin not only relieves heat, but also promotes the growth of new flesh, relieves pain, and kills worms. In the case of pine needles, it is recorded that they have the effect of prolonging the lifespan, and pine tree branches cure numbness in the legs and alleviate joint pain [15]. Recent studies have also demonstrated additional pharmacological activities such as anti-fungal, antioxidant, anti-cancer, anti-bacterial, anti-melanogenic, anti-atopic dermatitis, and anti-viral effects [16]. In this study, ethanol extract from pine tree branches (Pd-EE) was prepared to study whether this plant has anti-inflammatory activity in gastritis and lung injury. Because, in the case of gastritis, it is considered a prognostic lesion of gastric cancer, which is common and has a high mortality rate worldwide [17,18]. ALI was also confirmed because the lung, along with gastric tissue, was chosen as an important organ among the organs to fight various pathogens as well as other external factors [19]. In addition, the molecular mechanism of Pd-EE-mediated anti-inflammatory action was also elucidated.

## 2. Results

### 2.1. Pd-EE Alleviates the Inflammatory Response in Both In Vitro and In Vivo Models

**Figure 1:**
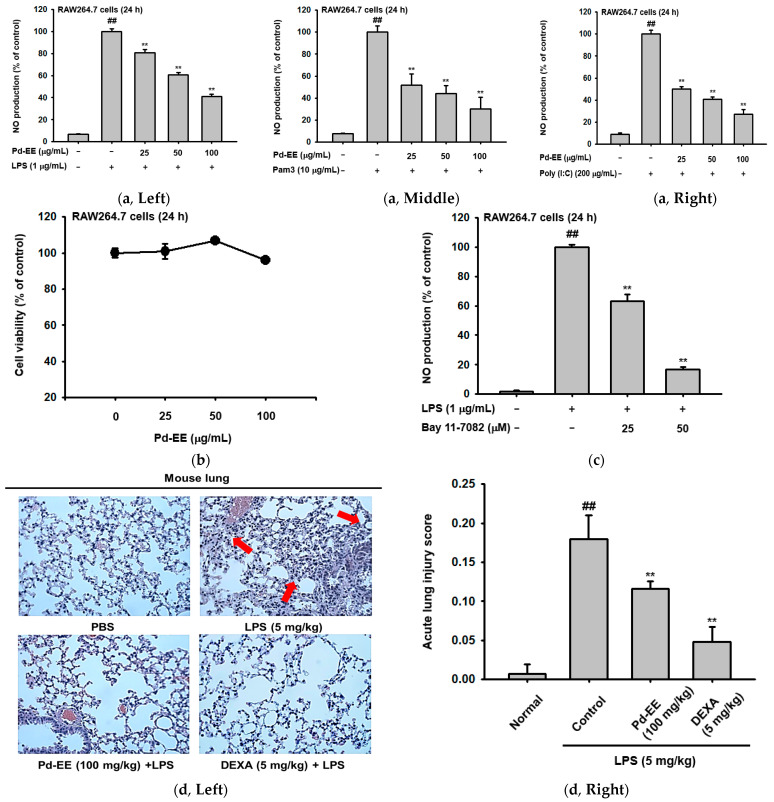

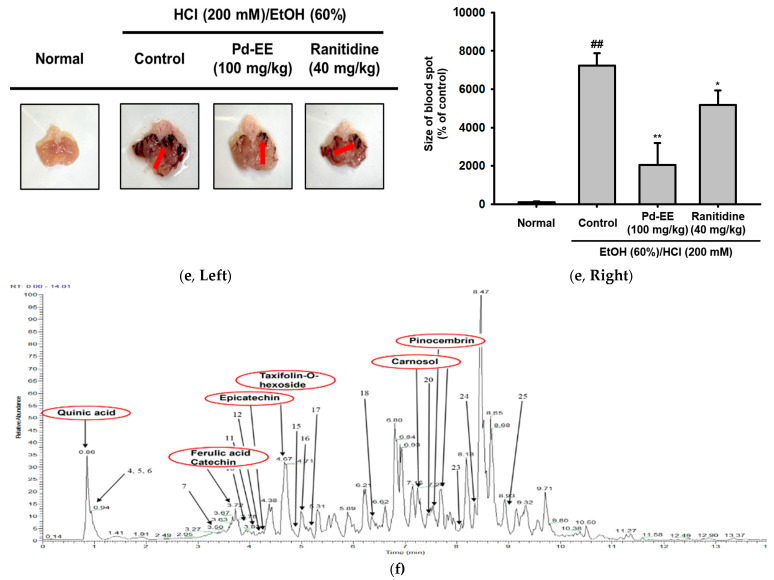
Anti-inflammatory effects of Pd-EE in in vitro and in vivo models. (**a**) NO production as percentage of the control when Pd-EE (0–100 μg/mL) was administered: (**a**, **left panel**) induced by LPS (1 μg/mL); (**a**, **middle panel**) induced by Pam3CSK4 (10 μg/mL); and (**a**, **right panel**) induced by Poly (I:C) (200 μg/mL). (**b**) The cytotoxicity of Pd-EE for RAW264.7 cells treated only with Pd-EE (0–100 μg/mL). (**c**) NO production when Bay 11-7082 (0–50 μM) was administered after LPS (1 μg/mL) induction. (**d**, **left panel**) Microscopic images of H&E staining for lung tissues in an in vivo acute lung injury model, achieved by intranasal injection of LPS (5 mg/kg). Pd-EE (100 mg/kg) and dexamethasone (5 mg/kg) were orally injected. (**d**, **right panel**) Acute lung injury scores for each lung injury experimental group based on H&E staining data. (**e**, **left panel**) Photographs of the stomachs removed immediately after in vivo acute gastritis experiment. After oral injections of Pd-EE (100 mg/kg) or ranitidine (40 mg/kg), the mice were sacrificed after 1 h of induction with HCl/EtOH. The stomachs were removed, and then photographed. (**e**, **right panel**) The degree of damage to the stomach tissue quantified using the ImageJ program. (**f**) LC-MS analysis of the compounds present in Pd-EE. ^##^ *p* < 0.01 compared to LPS-untreated group; ** *p* < 0.01 and * *p* < 0.05 compared to the LPS-treated group for the compound-treated group.

First, an NO assay was performed to determine whether the inflammatory response was inhibited by Pd-EE. NO is a hallmark of inflammatory response, and LPS (which interacts with TLR4), Pam3CSK4 (which interacts with TLR1/2), and poly I:C (which interacts with TLR3) were used to stimulate TLRs in RAW264.7 cells to induce NO production [20,21,22]. When TLR stimulators were administered, the produced NO decreased in a concentration-dependent manner with Pd-EE dosage (0–100 μg/mL). The Pd-EE reduced NO production by 30% at 100 μg/mL (Figure 1a). We also confirmed that Pd-EE did not have cytotoxicity against RAW264.7 cells at these concentrations (25–100 μg/mL; Figure 1b). Additionally, the NO inhibitory ability of Pd-EE was compared with that of the control compound, Bay 11-7082 (0–50 μM; Figure 1c).

The anti-inflammatory effects of Pd-EE could also be found in an in vivo mouse model. In the in vivo acute lung injury model, H&E staining data for the right lobe of the extracted lung were confirmed for histological evaluation. Scores were measured after observation using a microscope for the upper, lower, left, and right fields of each lobe of each group. The indices used for scoring included the number of neutrophils in the alveolar space and in the interstitial space, the number of hyaline membranes, and alveolar septal thickening. The scoring system was applied with reference to the report from American Thoracic Society Documents [23] (Table 1). The group in which acute lung injury was induced with LPS (5 mg/kg) had an 18-fold increase in score compared to the normal group that did not receive induction or drug treatment. In the group treated with Pd-EE (100 mg/kg) in addition to the LPS (5 mg/kg) induction condition, the measured score was lower than that of the control group (Figure 1d).

To check whether Pd-EE exhibits anti-inflammatory effects in acute gastritis as well as acute lung injury, the stomachs of mice with HCl/EtOH-induced lesions were observed. Visually, when ulcerative lesions were compared by group, the degree of damage was less in the groups treated orally with Pd-EE (100 mg/kg) or dexamethasone (5 mg/kg) compared to the group induced only with HCl/EtOH (200 mM). When the blood spots were measured numerically, we confirmed that the inflammatory lesions were reduced in the drug-treated groups as well (Figure 1e). Therefore, we found that Pd-EE has anti-inflammatory effects both cytologically and histologically.

These anti-inflammatory effects were also demonstrated by LC-MS analysis of Pd-EE. Through this analysis, quinic acid, its multiform, ferulic acid, catechin, epicatechin, taxifolin, its multiform, carnosol, and pinocembrin were observed for Pd-EE (Figure 1f). The detected metabolites are known to have anti-inflammatory effects [24,25,26,27,28,29]. Therefore, this also provides supporting evidence for Pd-EE to have anti-inflammatory effects.

### 2.2. Anti-Inflammatory Activity of Pd-EE at the Level of mRNA Expression

**Figure 2:**
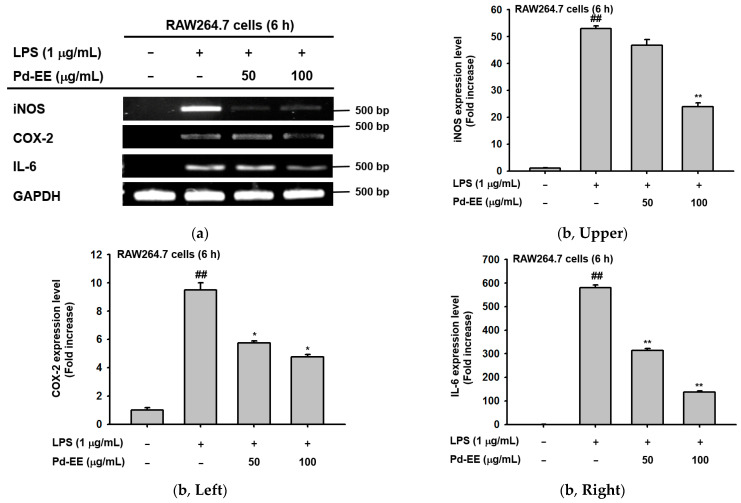

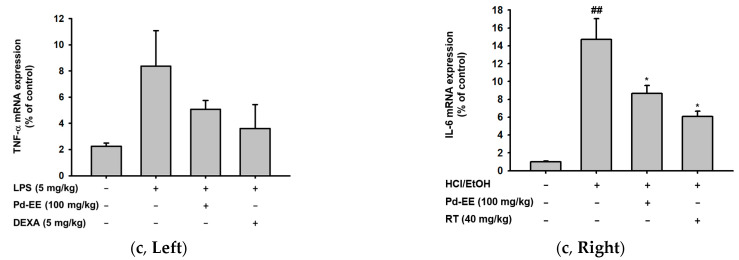
Inhibitory effects of Pd-EE on the mRNA expression levels of inflammation-related genes. (**a**) After pre-treatment with Pd-EE (0–100 μg/mL) and induction with LPS (1 μg/mL) for 6 h, mRNA expression levels of iNOS, COX-2, and IL-6 were confirmed. (**b**) Quantitative RT-PCR results for the genes identified in (**a**): (**b**, **upper panel**) mRNA expression level of iNOS; (**b**, **left panel**) mRNA expression of COX-2; (**b**, **right panel**) mRNA expression of IL-6. (a, b) GAPDH was used as a control gene. (**c**, **left panel**) The expression level of TNF-α measured from mRNA extracted with TRIzol solution by grinding the excised lung tissues after acute lung injury. (**c**, **right panel**) The expression level of IL-6 measured from mRNA extracted with TRIzol solution by grinding the excised stomach tissues after acute gastritis. ^##^ *p* < 0.01 compared to LPS-untreated group; ** *p* < 0.01 and * *p* < 0.05 compared to the LPS-treated group for compound treated group.

Next, based on the results of the NO assay, we investigated the changes in the mRNA expression levels of iNOS that occurred with or without Pd-EE treatment. Under the Pd-EE treatment condition (50–100 μg/mL), the mRNA expression of iNOS was reduced to such a degree that the band faded. The mRNA expression levels of COX-2 and IL-6, along with iNOS, were decreased in the same pattern (Figure 2a). Pd-EE (50–100 μg/mL) was found to decrease mRNA expression levels of these three factors in quantitative RT-PCR as well as semi-quantitative RT-PCR (Figure 2b). The same results were shown for in vivo ALI and gastritis in the reduction of mRNA expression of inflammation-related factors. We also evaluated the mRNA expression levels of TNF-α, which is known as an indicator of acute lung injury [30]. Its mRNA level was increased in the control group but was decreased in the groups treated with Pd-EE (100 mg/kg) or dexamethasone (5 mg/kg) (Figure 2c). Lastly, when the mRNA expression levels in gastric tissue were measured in the acute gastritis model, we confirmed that the expression of IL-6 was decreased in the group injected orally with Pd-EE (100 mg/kg) and ranitidine (40 mg/kg) (Figure 2c). In this way, the ability of Pd-EE to inhibit inflammation at the mRNA expression level was also demonstrated in both in vitro and in vivo models.

### 2.3. Pd-EE Affects the Transcriptional Level from the Activity Level of Factors in the Inflammatory Pathway

**Figure 3:**
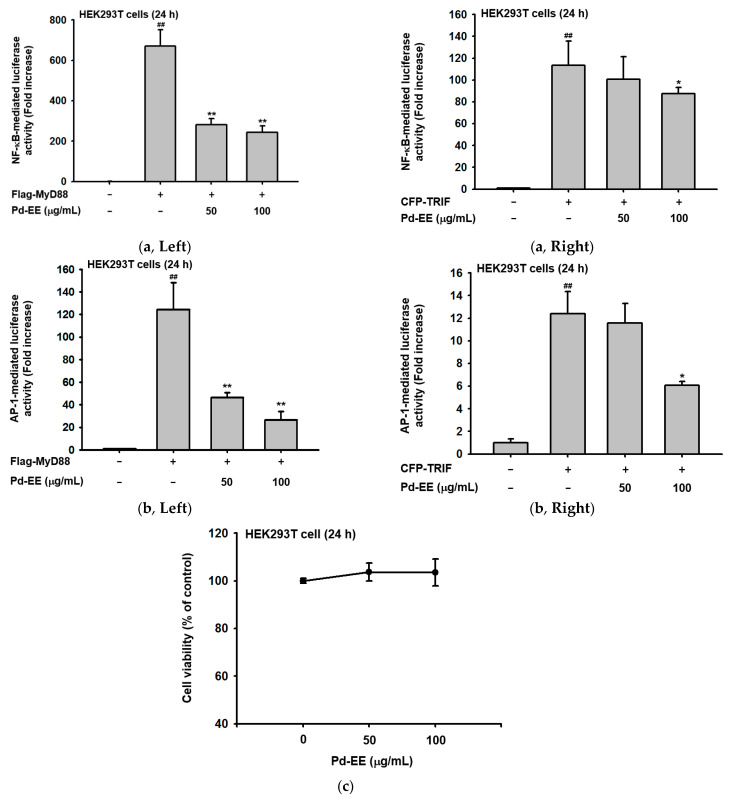

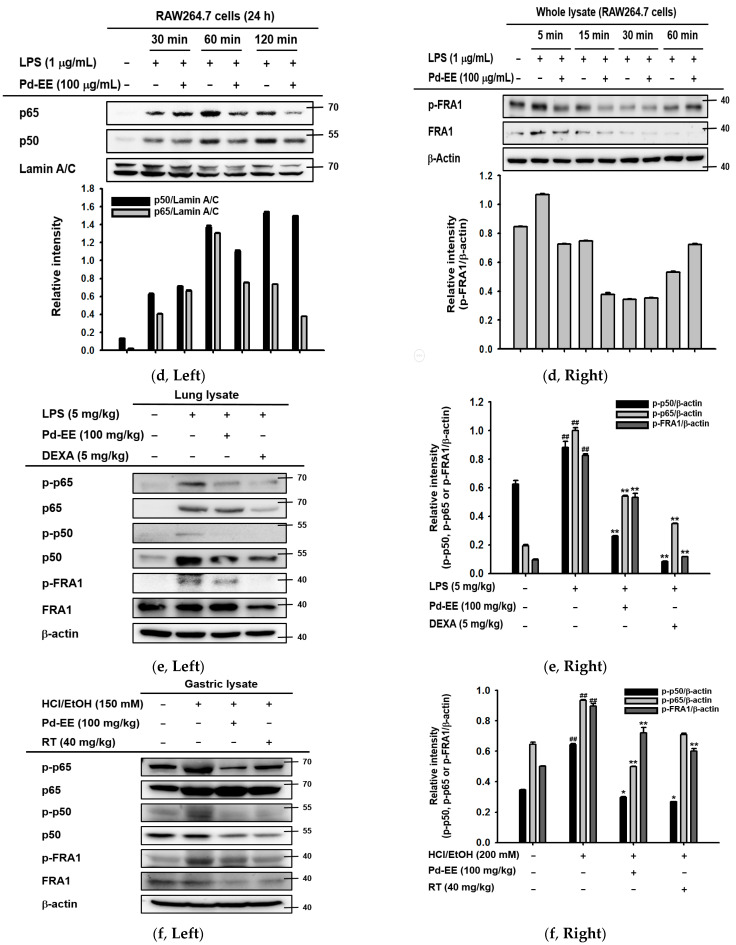

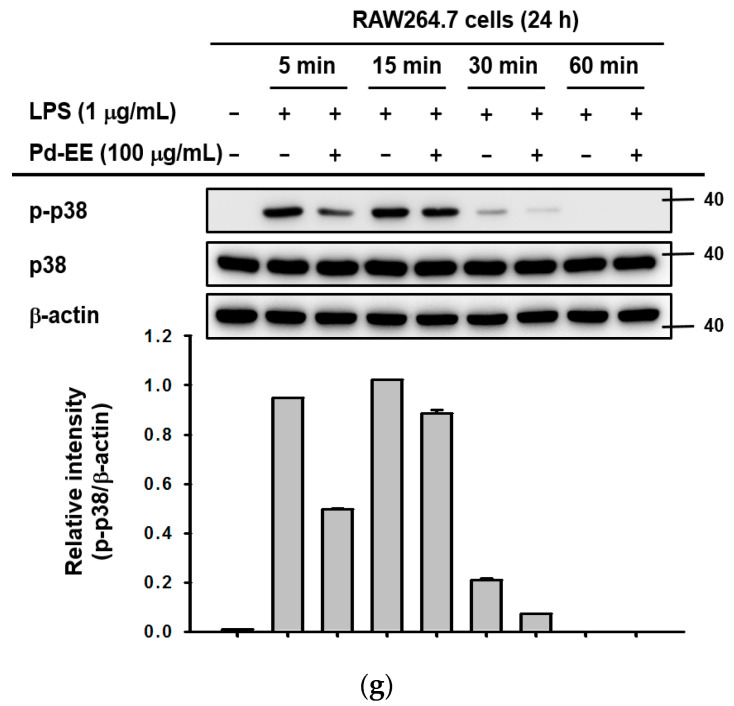
Suppressive effects of Pd-EE on factors in the inflammatory pathways. (**a**) HEK293T cells were transfected with (**a**, **left panel**) FLAG-MyD88 or (a, right panel) CFP-TRIF in addition to the NF-κB-luc plasmids. (**b**) HEK293T cells were transfected with (b, left panel) FLAG-MyD88 or (b, right panel) CFP-TRIF in addition to the AP-1-luc plasmids. Afte transfection, Pd-EE (0–100 μg/mL) was administered. Transcription factor-mediated luciferase activity was measured with a luminometer using luciferin. The β-gal plasmid was used for normalization. (**c**) Cytotoxicity to HEK293T cells was confirmed through formazan formation after only Pd-EE (100 μg/mL) was administered. (**d**, **left panel**) RAW264.7 cell lysate was obtained by administering Pd-EE (100 μg/mL) first, then LPS (1 μg/mL) for 30 min, 1 h, and 2 h. This was used to check the protein expression level by decomposing the lysate with a lysis buffer. First, the expression levels of total proteins of p65 and p50 were checked by the nuclear fractionation method. Lamin A/C was used as a control protein. (**d**, **right panel**) Phosphorylation level of FRA1 and expression of total protein were also confirmed after LPS was administered, and the control protein used at this time was β-actin. The band intensities were also calculated and compared through the ImageJ program for p50/Lamin A/C, p65/Lamin A/C, and *p*-FRA1/β-actin. (**e**, **left panel**) For the factors identified in (**d**), the protein levels were confirmed by grinding the frozen lung tissue of the ALI model. The phosphorylation levels of p65, p50 and FRA1 and the total protein expression level were confirmed. For this, the corresponding primary antibody was administered overnight, and the secondary antibody was applied for 2 h. (**e**, **right panel**) Band intensities were also compared for β-actin. (**f**) As in (**e**), the protein expression level and band intensities were also measured in the frozen gastric tissue in the acute gastritis model. (**g**) The phosphorylation level of p38 and the total protein level were confirmed in the same way as the expression of FRA1. ^##^ *p* < 0.01 compared to MyD88 or TRIF or LPS-untreated group; ** *p* < 0.01 and * *p* < 0.05 compared to the MyD88 or TRIF or LPS-treated group for compound treated group.

After we confirmed that Pd-EE inhibits the mRNA expression of inflammation-related genes, we conducted an experiment to determine whether the activity of transcription factors is also regulated by Pd-EE. When the NF-κB pathway and the AP-1 pathway were induced with the adapter molecules TRIF or MyD88, the activities of the corresponding transcription factors were reduced under the Pd-EE treatment conditions. At a concentration of 50 μg/mL, the activity of transcription factors was decreased, and at a concentration of 100 μg/mL, it was further reduced (Figure 3a–b). Pd-EE (0–100 μg/mL) also did not show any cytotoxicity against the HEK293T cells used when the luciferase assay was performed (Figure 3c). Based on these results, we observed that the protein expression levels of p50 and p65, which are subunits of the NF-κB family [31], were reduced when Pd-EE (100 μg/mL) was administered more than 60 min after LPS induction (Figure 3d). In the case of AP-1, the phosphorylation level of FRA1, which is known to increase the activity of this transcription factor, was reduced under the Pd-EE treatment conditions [32]. Its phosphorylation decreased between 5 and 30 min after LPS treatment (Figure 3d).

The decreases in protein expression levels and phosphorylation levels of the factors (p50, p65, and FRA-1) identified in vitro were also shown in the in vivo model (Figure 3e–f). When affirmed from the tissues of the in vivo acute lung injury and gastritis models, the protein expression levels and phosphorylation of these factors (p50, p65, and FRA-1) were decreased in the tissues of the group injected orally with Pd-EE (100 mg/kg) more than in the tissues of the group induced only with LPS or HCl/EtOH. This decreasing trend was the same as the trend observed in the tissues of the group orally injected with the control drugs (Dexa [5 mg/kg] or RT [40 mg/kg]) used in the experiments (Figure 3e–f). Additionally, the phosphorylation level of p38, known as an upstream player of FRA-1 [33], was also reduced by Pd-EE from 5 to 30 min after LPS treatment (Figure 3g). Hereby, it was demonstrated that Pd-EE affects the activities of inflammation-regulatory transcription factors.

## 3. Discussion

The extract (*Pinus densiflora* Siebold and Zucc. ethanol extract) evaluated in this study was extracted from the branches of pine trees; according to traditional knowledge, some parts of pine trees are known to have been used to reduce pain, reduce fever, and kill worms. Based on this fact, research on whether Pd-EE has anti-inflammatory effects was conducted both in vitro and in vivo.

First, Pd-EE decreased the NO produced when macrophages were stimulated by LPS (reacts with TLR4), Pam3CSK4 (reacts with TLR1/2), and Poly (I:C) (reacts with TLR3) [34] (Figure 1a). NO is a mediator that induces inflammation due to its excessive production in the pathogenesis of inflammation [21]. Through this, we showed that Pd-EE has anti-inflammatory effects in vitro. This efficacy was also demonstrated by a comparison with BAY 11-7082 (Figure 1c), which is known to have anti-inflammatory activity as an inhibitor of κB kinase [35]. The inhibition of NO production by Pd-EE was observed at the transcriptional level, since iNOS expression was attenuated (Figure 1). In fact, the activity of inflammation-regulatory transcription factors NF-κB and AP-1 was strongly downregulated (Figure 2), implying that these transcription factors could be targeted by Pd-EE, as shown in the cases of other medicinal plants such as *Olea europaea*, *Phyllanthus acidus*, *Euodia pasteuriana*, *Panax ginseng*, and *Alisma canaliculatum* [36,37,38,39].

Pd-EE not only reduced the number of neutrophils and the number of hyaline membranes, but also decreased alveolar septal thickening in an in vivo acute lung injury model. The formation of hyaline membranes or alveolar septal thickening are histological characteristics of acute lung injury [40], so Pd-EE alleviated the lung injury (Figure 1d). Additionally, in the acute gastritis model, it was visually confirmed that the degree of tissue damage was reduced (Figure 1e). These results strongly suggest that Pd-EE can ameliorate acute symptoms of inflammation in stomach and lung. The inhibitory potency of Pd-EE in gastritis conditions was higher than those of ethanol or methanol extracts of *Panax ginseng*, *Sorbaria kirilowii*, *Potentilla glabra* and *Viburnum pichinchense* [41,42].

In LC-MS analysis conducted to find the evidence for the anti-inflammatory abilities of Pd-EE, many inflammation-related metabolites were recognized in Pd-EE (Figure 1f). These include quinic acid, which is known to inhibit vascular inflammation [24]; ferulic acid, which has anti-inflammatory and antioxidant properties [25]; and catechin, which has anti-inflammatory and antimicrobial effects [26]. Taxifolin, which has anti-inflammatory properties [27]; carnosol, which has anti-inflammatory and anticancer effects [28]; and pinocembrin, which inhibits allergic airway inflammation [29], were also found.

Next, the mRNA expression levels of COX-2, IL-6, and TNF-α were reduced in the Pd-EE-treated and Pd-EE-injected conditions (Figure 2). The COX-2 is an enzyme that generates prostaglandin E, which is mediator-produced in the inflammatory process, and TNF-α and IL-6 activate macrophages and increase the expression levels of other inflammation-related cytokines to promote inflammatory responses [43]. Therefore, the decrease in the mRNA levels of these factors can be considered as a decrease in the inflammation. The activities of NF-κB and AP-1, which play important roles in biological and pathological pathways including inflammation, were also reduced by Pd-EE (Figure 3a–b) [44]. NF-κB and AP-1 are transcription factors that can regulate the expression of pro-inflammatory enzymes and cytokines by binding to their target DNA [45,46]. The results of Figure 3a,b make sense with the previous results. Accordingly, we confirmed that the total protein levels and phosphorylation of p50 and p65, which are components of NF-κB [47], were reduced by Pd-EE (Figure 3d–f). In the case of FRA1, an Fos member of AP-1 [48], it was demonstrated that phosphorylation was reduced by Pd-EE (Figure 3d–f). Phosphorylation of p38, a type of mitogen-activated protein kinase (MAPK) known to regulate transcription factor AP-1 [49], was also reduced when Pd-EE was administered (Figure 3g). Therefore, these results might suggest that p38 of MAPK might be an upstream target of Pd-EE contributing to its AP-1 pathway inhibitory activity. Considering that *Saururus chinensis* and *Barringtonia augusta* inhibited TAK1, an upstream enzyme to activate MAPK and AP-1, and *Cerbera manghas* reduced the enzyme activity of JNK, one of MAPKs [50,51,52], Pd-EE might block one of the upstream enzymes to activate AP-1 pathway. In addition, it is expected that Pd-EE can inhibit one of the upstream kinases regulating NF-κB pathway, since Src, Syk, PDK1, AKT, and IKK were targeted by several NF-κB-inhibitory plants including *Olea europaea*, *Mycetia cauliflora*, *Lilium brownii*, and *Chrysanthemum indicum* [53,54,55,56]. Whether these enzymes including p38 are directly targeted by Pd-EE will be further explored using a kinase assay and an overexpression strategy with candidate target gene(s).

## 4. Materials and Methods

### 4.1. Materials and Reagents

Pd-EE (NIBRGR0000433437) obtained from the Wildlife Natural Products Bank and voucher specimen (KONPVP0000373423) is preserved at the herbarium of the National Institute of Biological Resources (Incheon, Korea). LPS (Escherichia coli 0111:B4), Pam3CSK, poly I:C, polyethyleneimine (PEI), dexamethasone, and ranitidine were purchased from Sigma Chemical Co. (St. Louis, MO, USA). RAW264.7 cells (ATCC number TIB-71) and HEK293 cells (ATCC number CRL-1573) were purchased from the American Type Culture Collection (ATCC; Rockville, MD, USA). Fetal bovine serum (FBS), Roswell Park Memorial Institute (RPMI) 1640 medium, Dulbecco’s Modified Eagle’s medium (DMEM), phosphate buffered saline (PBS), TRIzol reagent, and Opti-MEM Reduced Serum Medium were purchased from Gibco (Grand Island, NY, USA). In addition, p38, p-p38, p50, p-p50, p65, p-p65, FRA1, p-FRA1, and Lamin A/C were purchased from Cell Signaling Technology (Beverly, MA, USA), and β-actin was purchased from Santa Cruz Biotechnology, Inc. (Santa Cruz, CA, USA).

### 4.2. Cell Culture and Extract Preparation

RAW264.7 cell lines were cultured in RPMI 1640 and sub-cultured once every two days. HEK293T cell lines were cultured in DMEM and were sub-cultured once every three days. Both media contained inactivated FBS and antibiotics (penicillin and streptomycin). The stock solution of Pd-EE (100 mg/mL) was dissolved in dimethyl sulfoxide (DMSO) and diluted to 0–100 μg/mL using cell culture media when it is treated. For in vivo animal models, Pd-EE was resolved in 0.5% sodium carboxymethyl cellulose or PBS to prepare the critical concentration (100 mg/kg).

### 4.3. Nitric Oxide (NO) Production Assay

RAW264.7 cells were plated in 96-well plates at a density of 1 × 10^6^ cells/mL. Then, 100 μL of cells per well were incubated for about 20 h in an incubator at 5% CO2 and 37 °C. Pd-EE was diluted to 25–100 μg/mL using media and 50 μL was administered to the compound group. For the normal and control groups, DMSO was resolved in media and administered at the same concentration. After 30 min, an LPS stock solution (2 mg/mL) was diluted to 1 μg/mL and administered to the control and compound groups. Pam3CSK4 (10 μg/mL) or poly(I:C) (200 μg/mL) was also administered to the control and compound groups. After an induction of 24 h, the absorbance at 540 nm was measured after mixing a 1:1 ratio of the Griess solution with 100 μL of the supernatant [57].

### 4.4. Cell Viability Assay

Cells were incubated for 4 h with 10 μL of MTT solution (5 mg/mL) in an incubator (5% CO_2_ and 37 °C). To stop the reaction and dissolve the formazan crystals, we added 100 μL of MTT stopping solution, and then the absorbance was checked at 570 nm.

### 4.5. mRNA Expression Level Analysis by Semi-Quantitative and Quantitative Reverse Transcription Polymerase Chain Reaction

LPS was diluted to 1 μg/mL and administered to RAW264.7 cells pre-treated with Pd-EE for 30 min. After 6 h, all medium was suctioned, and the total RNA was extracted from the remaining cells with TRIzol reagent and stored at −70 °C. Using this mRNA, cDNA synthesis and RT-PCR were performed as previously described [58]. Quantification of the mRNA expression levels of iNOS, TNF-α, COX-2, interleukin (IL)-1β, IL-6, and GAPDH was performed by semi-quantitative and quantitative RT-PCR [41]. In the case of semi-quantitative RT-PCR, mRNA could be quantified using EtBr, and in the case of quantitative RT-PCR, mRNA could be traced through SYBR Green. The primers used are presented in Table 2.

### 4.6. Luciferase Reporter Gene Assay

HEK293T cells were plated at a density of 2 × 10^5^ cells/mL and co-transfected with plasmids such as NF-κB-Luc or AP-1 Luc. The transfection was processed using PEI. The plasmids were placed in Opti-MEM media, stabilized for 15 min, and then mixed with PEI solution. After an additional 15 min of stabilization, the plasmids were administered to cells for 24 h. Then, Pd-EE was administered for 24 h. The next day, the cells were lysed by freezing at −70 °C and thawing. The luciferase reporter activity was measured by checking the luminescence after luciferin was added [59,60]. The activity was also normalized to that of β-galactosidase.

### 4.7. Whole Lysate Preparation and Western Blotting Assay

RAW264.7 cells treated with Pd-EE and LPS were washed and collected in tubes using PBS. After centrifugation (8000 rpm, 5 min), the cell pellets were lysed with lysis buffer r (20 mM Tris-HCl, pH 7.4; 2 mM EDTA; 2 mM ethyleneglycotetraacetic acid; 1 mM DTT; 50 mM β-glycerol phosphate; 0.1 mM sodium vanadate; 1.6 mM pervanadate; 1% Triton X-100; 10% glycerol; 10 μg/mL aprotinin; 10 μg/mL pepstatin; 1 μM benzamide; and 2 μM PMSF). After being centrifuged once more, the supernatants were used for western blotting assays. Immunoblotting was performed with specific antibodies, as reported previously [61,62].

### 4.8. EtOH/HCl-Induced Gastritis Mouse Model

Five-week-old male ICR mice (5 mice/group) were orally treated with Pd-EE (100 mg/kg) or ranitidine (40 mg/kg) three times per day for two days. At the third day, the gastritis was induced with EtOH/HCl (300 μL of 60% ethanol in 200 mM HCl) at 8 h after the final oral injection. The mice were anesthetized and sacrificed using isoflurane, and their stomachs were extracted. The stomachs were washed with PBS several times and photographed. Photographs of the injured lesions were analyzed using ImageJ software (ImageJ bundled with 64-bit Java 1.8.0_172). Then, the stomach tissue was ground with liquid nitrogen and stored at −70 °C until they were used. When used in western blotting assays, the tissues were lysed in lysis buffer, and when mRNA levels were checked, they were lysed by TRIzol reagent. The approval number received for this experiment is as follows (SKKUIACUC2020-09-48-1).

### 4.9. LPS-Induced Acute Lung Injury Mouse Model

Six-week-old male C57BL/6 J mice (5 mice/group) were orally treated with Pd-EE (100 mg/kg) or dexamethasone (5 mg/kg) 3 times per day (10 AM, 4 PM, and 6 PM). Before the final oral injection, they were treated with 50 μL of LPS (5 mg/kg) two times every 30 min by intranasal administration. After 16 h of induction, the lungs were removed and divided into two parts. The left lobes were fixed in 3.7% paraformaldehyde for 1 day. The fixed tissues were embedded in paraffin. After sectioning (4 μm), they were stained with hematoxylin and eosin and examined for parameters of tissue injury under a microscope (4×, 10×, and 20×). In the case of the left lobes, they were stored at −70 °C. The next day, the tissues were ground with liquid nitrogen. Then, half of the ground tissue was suspended in lysis buffer and used for protein level analysis, and the other half was used for mRNA expression level confirmation using TRIzol reagent. The approval number received for this experiment is as follows (SKKUIACUC2021-05-58-1).

### 4.10. LC-MS Analysis

Each sample (10 mg/mL) was dissolved in 80% ethanol for UHPLC-LTQ-Orbitrap-MS/MS analysis. This was performed using a UHPLC system equipped with a Vanquish binary pump H system (Thermo Fisher Scientific, Waltham, MA, USA) coupled with an auto-sampler and column compartment. The chromatographic separation was performed on a Phenomenex KINETEX^®^ C18 Column (100 mm × 2.1 mm, 1.7 μm; Torrance, CA, USA) at a column oven temperature of 40 °C. The mobile phases consisted of 0.1% formic acid in water (A) and in acetonitrile (B). The 5% of solvent B was maintained initially for 1 min, followed by a linear increase to 100% solvent B over 9 min, and then was sustained at 100% solvent B for 1 min, with a gradual decrease to 5% solvent B over 3 min. The total run time was 14 min. The flow rate was 0.3 mL/min, and the injection volume was 5 μL. The mass spectra were obtained using electrospray ionization in both negative and positive modes within a range of m/z of 100–1500. The metabolites were identified by comparing their retention times and mass fragment patterns with in-house library data, references, and several databases, such as the National Institutes of Standard and Technology Library (v.2.0, 2011, FairCom, Gaithersburg, MD, USA), the Dictionary of Natural Products (v.16:2, 2007, Chapman and Hall, USA), Wiley 8, and the Human Metabolome Database (http://www.hmdb.ca/ (accessed on 3 March 2021)).

### 4.11. Statistical Analysis

The overall significance of each result was first evaluated through one-way ANOVA and then the Mann–Whitney U test was further used. *p*-values less than 0.05 were considered statistically significant. All data are presented as means ± standard errors.

## 5. Conclusions

**Figure 4:**
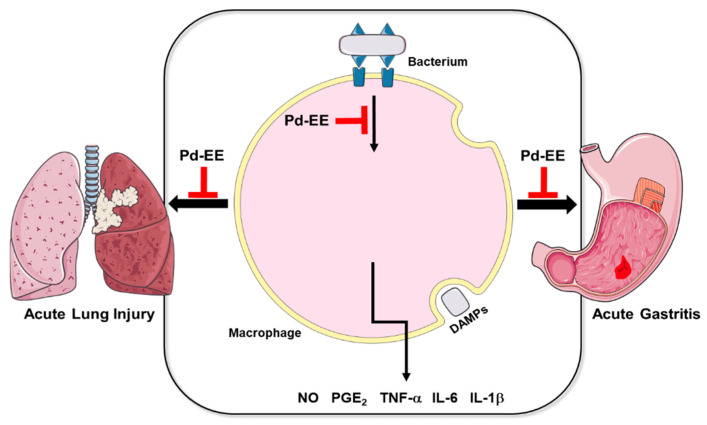
Scheme of Pd-EE’s anti-inflammatory activity.

We have demonstrated that Pd-EE can attenuate acute inflammatory damage by the suppression of both the AP-1 and NF-κB pathways in vitro and in vivo (Figure 4). These findings suggest that Pd-EE can be considered a candidate for herbal medicine that can modulate the inflammatory symptoms in the stomach and lungs. However, for this purpose, it is necessary for us to investigate whether Pd-EE is also non-toxic or does not display any side effects. In addition, finding more precise targets for a full understanding of molecular mechanisms may help to expand the therapeutic efficacy of Pd-EE.

## Figures and Tables

**Table 1:** Lung injury scoring system used in analysis of H&E staining data.

Parameter	Score per Field		
	0	1	2
A. Neutrophils in the alveolar space	None	1–5	>5
B. Neutrophils in the interstitial space	None	1–5	>5
C. Hyaline membranes	None	3	>3
D. Alveolar septal thickening	< 2×	2–4 ×	>4×
Score = [(20 × A) + (14 × B) + (7 × C) + (2 × D)] / (number of fields × 100)			

**Table 2:** Primer sequences used in analysis of mRNA expression levels of mouse inflammatory genes by semiquantitative RT-PCR and quantitative PCR.

Gene	Direction	Sequences (5′ to 3′)
iNOS (semi-RT-PCR)	Forward Reverse	TGCCAGGGTCACAACTTTACA ACCCCAAGCAAGACTTGGAC
iNOS (qPCR)	Forward Reverse	GCCACCAACAATGGCAACAT TCGATGCACAACTGGGTGAA
COX-2 (semi-RT-PCR)	Forward Reverse	TCACGTGGAGTCCGCTTTAC TTCGACAGGAAGGGGATGTT
COX-2 (qPCR)	Forward Reverse	TTGGAGGCGAAGTGGGTTTT TGGCTGTTTTGGTAGGCTGT
IL-6 (semi-RT-PCR)	Forward Reverse	GCCTTCTTGGGACTGATGG TGGAAATTGGGGTAGGAAGGAC
IL-6 (qPCR)	Forward Reverse	AGCCAGAGTCCTTCAGAGAGA AGGAGAGCATTGGAAATTGGGG
TNF-α (semi-RT-PCR)	Forward Reverse	TGCCTATGTCTCAGCCTCTT GAGGCCATTTGGGAACTTCT
GAPDH (semi-RT-PCR)	Forward Reverse	GAAGGTCGGTGTGAACGGAT AGTGATGGCATGGACTGTGG
GAPDH (qPCR)	Forward Reverse	TGTTGAACGGATTTGGCCGTA ACTGTGCCGTTGAATTTGCC

## Data Availability

The data used to support the findings of this study are available from the corresponding author upon request.

## Sample Availability

Sample of the compound, Pd-EE is available from the authors.

## Abbreviations

TAK1	Transforming growth factor-β-activated kinase 1
NF-κB	Nuclear factor-κB
AP-1	Activator protein-1
MAPKs	Mitogen-activated protein kinases
JNK	c-Jun N-terminal kinase
PI3K	Phosphoinositide 3-kinase
PGE_2_	Prostaglandin E_2_
COX-2	Cyclooxygenase-2
IL-1β	Interleukin-1β
IL-6	Interleukin 6
TNF-α	Tumor necrosis factor alpha
MKK	Mitogen-activated protein kinase
*iNOS*	Inducible nitric oxide synthase
